# A survey of mHealth use from a physician perspective in paediatric emergency care in the UK and Ireland

**DOI:** 10.1007/s00431-021-04023-0

**Published:** 2021-03-25

**Authors:** Haiko Kurt Jahn, Ingo Henry Johannes Jahn, Wilhelm Behringer, Mark D. Lyttle, Damian Roland, On behalf of Paediatric Emergency Research United Kingdom and Ireland (PERUKI)

**Affiliations:** 1 Friedrich-Schiller-Universität Jena, Jena, Thüringen Germany; 2 Children’s Emergency Department, Royal Belfast Hospital for Sick Children, 274 Grosvenor Rd, Belfast, BT12 6BA UK; 3 School of Mechanical and Mining Engineering, The University of Queensland, Brisbane, QLD 4072 Australia; 4 Faculty of Medicine, Center of Emergency Medicine, Friedrich-Schiller-Universität Jena, Jena, Thüringen Germany; 5 Emergency Department, Bristol Royal Hospital for Children, Upper Maudlin Street, Bristol, BS2 8BJ UK; 6 Academic Department of Emergency Care, University of the West of England, Blackberry Hill, Avon, Bristol, BS16 1DD UK; 7 SAPPHIRE Group, Health Sciences, University of Leicester, Leicester, LE1 6TP UK; 8 Children’s Emergency Department, Royal Infirmary, Paediatric Emergency Medicine Leicester Academic (PEMLA) Group, Leicester, LE1 5WW UK

**Keywords:** Child health, Emergency medicine, Information science, Health service research, Paediatrics

## Abstract

**Abstract:**

There has been a drive towards increased digitalisation in healthcare. The aim was to provide a snapshot of current apps, instant messaging, and smartphone photography use in paediatric emergency care. A web-based self-report questionnaire was performed. Individual physicians working in paediatric emergency care recorded their personal practice. One hundred ninety-eight medical doctors completed the survey. Eight percent of respondents had access to institutional mobile devices to run medical apps. Eighty-six percent of respondents used medical apps on their personal mobile device, with 78% using Apple iOS devices. Forty-seven percent of respondents used formulary apps daily. Forty-nine percent of respondents had between 1–5 medical apps on their personal mobile device. Respondents who used medical apps had a total of 845 medical apps installed on their personal device, accounted for by 56 specific apps. The British National Formulary (BNF/BNFc) app was installed on the personal mobile device of 96% of respondents that use medical apps. Forty percent of respondents had patient confidentiality concerns when using medical apps. Thirty-eight percent of respondents have used consumer instant messaging services, 6% secure specialist messaging services, and 29% smartphone photography when seeking patient management advice.

**Conclusion:**

App use on the personal mobile devices, in the absence of access to institutional devices, was widespread, especially the use of a national formulary app. Instant messaging and smartphone photography were less common. A strategic decision has to be made to either provide staff with institutional devices or use software solutions to address data governance concerns when using personal devices.

**Table:** 

**What is Known:** *• mHealth use by junior doctors and medical students is widespread.* *• Clinicians’ use of instant messaging apps such as WhatsApp is the widespread in the UK and Ireland, in the absence of alternatives.*	
**What is New:** *• Personal mobile device use was widespread in the absence of alternatives, with the British National Formulary nearly universally downloaded to physicians’ personal mobile devices.* *• A third of respondents used instant messaging and smartphone photography on their personal mobile device when seeking patient management advice from other teams in the absence of alternatives.*

**Supplementary Information:**

The online version contains supplementary material available at 10.1007/s00431-021-04023-0.

## Introduction

The invention of mobile devices and apps has resulted in the development of mobile health (mHealth) [1, 2]. Coupled with technological advances, and near ubiquitous use of mobile devices in the population, mHealth can be used by both patients and clinicians. There are relatively few reports on its use in each group, and it is important to understand perceptions and use of mHealth from a physician perspective. Apps targeted at physicians have the potential to improve patient care by allowing immediate access to medical and health care information, improving decision making, reducing medical errors, and enhancing telemedicine capabilities [3–6]. The use of mHealth in paediatric emergency care for remote triage and video consultation has been reported [1, 7]; the use of mHealth in general in response to the COVID 19 pandemic has increased [8–10].

In paediatric emergency care, the use of digital aids to reduce human error, especially in prescribing, has been recognised [11]. Developers have shown apps to be superior in inotrope prescribing compared to using hardcopy formularies, with medical students outperforming specialists [3, 12].

The rapid and organic growth of mHealth technology continually creates new challenges [13] including patient information being exposed to online attacks, concerns regarding confidentiality, lack of quality control, new evidence overtaking available algorithms and guidance, and patient and physician acceptability [7].

The introduction of the European GDPR Regulation in 2018 has mainly focused on ensuring a high level of communication encryption when using email or mobile device technology to ensure confidentiality when patient identifiable information (e.g. images or electronic records) are shared or accessed [14–16]. Serious breaches of patient confidentiality have been reported from the use of personal instant messaging apps, which have led to the development of secure specialist messaging apps for mHealth [16, 17]; yet despite this, surveys suggest widespread use of consumer-oriented instant messaging apps in healthcare in Europe [18–22].

Strategy and recommendations for institutions on how to manage the necessary digital transformation in healthcare exist for example in the UK, Ireland, Austria and Switzerland [15, 23–26]. Others such as Germany focussed on creating an electronic patient dossier, containing basic patient data and medication plans that can be accessed by all health providers involved in the patients care [27].

The aim of this survey was to provide a snapshot of current mHealth use from a healthcare provider perspective in paediatric emergency care. We hypothesis if the use of mHealth is of benefit to physicians working in paediatric emergency care, then the use of mHealth will be widespread. Specific examples of this would be if a formulary apps is easier to use than other resources, then physicians would use formulary apps frequently, because they provide near instantaneous information at the bedside. Or if instant messaging and smartphone photography is less cumbersome then departmental camera use for medical photography, then physicians will default to using their personal device, especially if no other alternatives such as institutional mobile devices are provided.

## Method

The online survey was undertaken between 31/07/2017 and 14/01/2018, delivered via SurveyMonkey (www.surveymonkey.com). Survey content was derived by the lead researcher from previous literature examining mobile device and medical app use by clinicians [6, 28, 29], and refined iteratively by an expert panel to ensure content validity and reliability, and piloted. This survey investigated individual physician use of mHealth, i.e. the use of mobile devices and apps to aid patient management as part of a wider study of mHealth use in emergency care (adult and paediatric) in English and German speaking countries in Europe [13, 30, 31]. The survey was distributed via (i) Paediatric Emergency Research in the UK and Ireland 45 site leads (PERUKI) [32], a collaborative paediatric emergency research network, which includes mixed (adult and paediatric) and stand-alone paediatric Emergency Departments from urban and rural settings (for further information see https://www.peruki.org) [32], (ii) the Royal College of Emergency Medicine newsletter and website, and (iii) social media. The link to the survey was shared on the twitter feed of the authors. Respondents accessed the survey via the shared link. Questions included multiple selection and free text answers (Appendix: Survey Questionnaire). Data collected included basic demographic information, mobile device policy, Wi-Fi access, mobile device type, app types, app use, app communication, and percep of app use in clinical practice.

### Statistical analysis

All completed responses were included in the analysis, this included survey responses were respondents skipped individual questions. Partially completed responses were excluded as analysis of these responses showed no noteworthy differences to the completed responses. Microsoft Excel (Version 16.18) was used to undertake descriptive analysis of complete responses. Free text was analysed either for single word answers e.g. name of an app, or the lead author conducted thematic analysis to identify common themes. The thematic analysis followed Braun and Clarke’s six phases of thematic analysis [33]. Following familiarisation with the data set, the authors created codes for answers with similar topics such as “smartphone photography”. Initial themes were then generated to give meaning to the codes. These themes were then checked against the entire coded data set. Co-authors verified this process. These themes were then analysed.

### Ethics

This survey accessed clinicians via a research collaborative to assess their individual practice and therefore did not require formal ethics review according to the Framework for Health and Social Care Research (UK) [34]. Consent was implied by participation.

## Results

We received 198 complete and 45 incomplete survey responses of physicians involved in resuscitating children and working in paediatric emergency care from the UK and Ireland. Individual physicians from 37 out of the 45 PERUKI sites responded to the survey (supplementary table 1) with a mean of 3 and a median of 2 responses per site, further details regarding site demographic can be found at www.peruki.org. The majority of respondents were consultants and worked at a PERUKI site, and the mean age of respondents was 39 years. Demographics including institutional mobile device, Wi-Fi provision and guidance are listed in Table 1 (supplementary table 1 and 2)

**Table 1:** Demographics individual clinician survey

Question	Response (*n* =198)	%
**What is your gender?**		
• Male	105	53%
• Female	93	47%
**Could you select your region?**		
• England	129	65%
• Northern Ireland	27	14%
• Scotland	19	10%,
• Wales	13	7%,
• Republic of Ireland	10	5%,
**What is your role?**		
• Consultants (attending physician)	103	52%
• Trainee Doctors	94	47%
• General Practitioners	1	1%
**Are you aware of your institution’s mobile device policy?**		
• Aware of institution’s mobile device policy	101	51%
**Are you provided with an institutional mobile device to run Medical Apps?**		
• Provided with institutional mobile device	16	8%
**Do you use Medical Apps on your personal mobile device?**		
• Use medical apps on personal mobile device	169	86%
**Wi-Fi access**		
• Free Wi-Fi access	122	62%
• No Wi-Fi access	63	32%
• Limited Wi-Fi access (e.g. poor signal strength)	13	7%

### Individual physician app use

Eighty-nine percent (177/198) of respondents reported using their personal mobile device for web access daily at work. Forty-seven percent (94/198) reported using a formulary app daily on their personal mobile device (Table 2)

**Table 2:** Respondents use of personal mobile device

	Daily (*n*=198)	%	Weekly (*n*=198)	%	Rarely (*n*=198)	%
• Web access	177	89%	6	3%	9	5%
• Calendar, rota	137	69%	24	12%	11	6%
• Email access (work email)	133	67%	7	4%	13	7%
• Formulary/drug reference	94	47%	52	26%	25	13%
• Clinical score/calculator	55	28%	58	29%	51	26%
• Education (revision and learning)	46	23%	61	31%	56	28%
• Disease diagnosis or management	33	16%	56	28%	70	35%
• CPD (Continuing Professional Development), NHS eportfolio etc.	15	8%	75	38%	52	26%
• Procedure documentation	6	3%	14	7%	42	21%

One hundred seventy respondents reported using medical apps on their personal mobile device at work. Of these 78% (132/170) reported using Apple IOS devices and 22% (38/170) Android devices. Of these 1% (1/170) had 0 medical apps, 55% (83/170) had 1–5 medical apps, 32% (54/170) had 6–10 medical apps, and 19% (32/170) had more than 10 medical apps installed on their personal mobile device.

One hundred sixty-three respondents provided a total of 845 free text responses of the names of the medical apps installed on their personal devices, accounted for by 56 specific apps. The leading app category was “resuscitation” with 170 instances, followed by formulary apps with157 instances. The *BNF/BNFc* formulary app (96%, 157/163) was the only app in this category. This was followed by an antimicrobial guidance app *Microguide* (42%, 69/163), *PaediatricEmergencies* (38%, 63/163), *GrowthChart* (27%, 44/163) and the *NeoMate* (25%, 40/163) app (Table 3).

**Table 3:** Apps on the personal mobile device respondents

Topic	Name of app	*n*=163	%
Resuscitation	• Paediatric Emergencies	63	39%
	• Neomate	40	25%
	• Paeds ED	21	13%
	• Paeds Emergency Tools	14	9%
	• iResus	11	7%
	• PICU calculator	11	7%
	• Mersey burn calculator	5	3%
	• Medical Emergency	3	2%
	• APLS	2	1%
Formulary	• BNF	157	96%
Calculator	• MDCalc	39	24%
	• MedCalx	25	15%
	• MedCalc	19	12%
	• Calculator	19	12%
	• Clinicalc	13	8%
	• grace calculator	10	6%
	• drug dose calculator	5	3%
	• Medical calculator	4	2%
Reference	• NICE	17	10%
	• Oxford handbook of clinical medicine	14	9%
	• Oxford handbook of emergency medicine	14	9%
	• Uptodate	14	9%
	• BMJ best practice	13	8%
	• RCH Melbourne clinical practical guidelines	11	7%
	• Medscape	9	6%
	• 3D Brain	4	2%
	• Neonatal Intensive Care	3	2%
	• Patient.co.uk	3	2%
	• Wikipedia	2	1%
	• Ganz	2	1%
	• Traumapedia	2	1%
Disease/task specific	• Growth Charts	44	27%
	• BiliApp	18	11%
	• POPS	11	7%
	• Med Gas Log	7	4%
	• NICU tools	7	4%
	• PediaBP	5	3%
	• Marispan	3	2%
	• My haemophilia centre	1	1%
Anti-microbial Guidance	• Microguide	69	42%
	• Alderhey antibiotic guidelines	11	7%
	• Mersey antibiotic guidelines	4	2%
Local/ Institutional	• Induction	20	12%
	• HANDi app	8	5%
	• HEFT EMapp	6	4%
	• NCHD.ie	5	3%
	• RBHSC app	5	3%
	• RLEMH	2	1%
	• EMed.ie	1	1%
Education	• SimMon	10	6%
	• EM:RAP	6	4%
	• BMJ OneExamination	5	3%
	• SCCM	4	2%
	• RCEM learning	3	2%
	• kaizen/eportfolio	3	2%
	• RCPCH CPD	2	1%
	• foam (free open access medical education)	2	1%
Communication	• mobileiron	14	9%

### Harm and perception of medical app use

No respondent reported any adverse events or patient harm from mobile device or medical app use as part of patient care. Seventy-two percent (144/198) of respondents perceived the use of medical apps as a positive development.

Fifty-nine percent (116/198) of respondents selected that they as physicians perceived app use during consultations as acceptable. When asked for their opinion on how their patients perceived app use during consultation, 46% (91/198) selected acceptable and 46% (92/198) selected unacceptable, unprofessional or showing lack of knowledge (Table 4). One hundred thirty-eight respondents provided free text responses related to patient comments on medical app use (supplementary table 3). Forty-five percent (89/198) reported that they received no comments from patients, and stated that this was most likely because they always explained app use to patients prior to use.

**Table 4:** Perception of app use

	Perception: Unprofessional, rude, shows lack of knowledge (*n*=198, %)	Perception: Acceptable (*n*=198, %)	Perception: Professional (*n*=198, %)	Other (*n*=198, %)
Physician perception of medical app use	44, 22%	116, 59%	28, 14%	10, 5%
Analysis of responses	33 unprofessional 8 rude 3 shows lack of knowledge	84 acceptable 32 acceptable if explained	28 professional	7 not in front of patients 3 mixed
Patient and carer perception of medical app use from physicians’ perspective	91, 46%	92, 46%	4, 2%	11, 6%
Analysis of responses	44 unprofessional 34 shows lack of knowledge 13 rude	64 acceptable 28 acceptable if explained	4 professional	9 mixed 2 unsure

There was an almost equal divide amongst respondents in relation to confidentiality concerns when using medical apps; 40% (79/198) had concerns, 31% (61/198) had no concerns, and 29% (58/198) were unsure. Sixty-six respondents highlighted their patient confidentiality concerns in additional free text responses (supplementary table 4)

### Mobile device use for communication

38% (76/198) of respondents reported prior use of consumer instant messaging services, 6% (12/198) secure specialist messaging services, 29% (57/198) text messaging (SMS), and 29% (56/198) smartphone photography when seeking patient management advice from colleagues or other specialities (supplementary table 5).

The leading reported reason for using smartphone photography and instant messaging as a communication tool on personal devices was convenience and ease of use 22% (44/198), followed by a lack of institutional alternatives in 14% (28/198). Six percent (12/198) reported that they only use smartphone photography and instant messaging on institutional devices.

For accessing medical apps (e.g. formulary app) 72% (142/198) preferred personal devices, compared to 10% (19/198) who preferred institutional devices. In contrast, for medical photography institutional devices were preferred by 40% (78/198), whilst 9% (18/198) of respondents used these functions on personal devices.

Of respondents using smartphone photography and instant messaging, 29% (57/198) anonymised patient details as an added security feature (i.e. either no patient identifier or initials only included). A minority did not anonymise photographs (4%, 8/198) or instant messages (3%, 6/198) sent from personal devices, citing end-to-end encryption (supplementary table 6)

### Barriers and drivers/enablers of app use

Eighty-five percent (169/198) of respondents reported barriers to medical app use, with the main barrier being the lack of internet or Wi-Fi connection. Ninety-one percent (181/198) of respondents reported enablers and drivers for medical app use, in which time saving and simplification of tasks were most frequently reported (Table 5 and supplementary table 7).

**Table 5:** Reported barriers and drivers/enablers of medical app use

	Barrier (*n*=169)	%	Driver/Enabler (*n*=181)	%
• Colleagues	19	11%	65	36%
• Institution	19	11%	31	17%
• Patients	15	9%	7	4%
• Technical Issues	60	36%	17	9%
• Internet or Wi-Fi Connection to run Medical App	101	60%, 101	64	35%
• Price*	58	34%	62	34%
• Time**	11	7%	109	60%
• Helpful***	7	4%	90	50%
• Other	26	15%	11	6%

*Price: barrier: high purchase cost; driver/enabler: free to download

**Time: barrier: prolongs task, driver/enabler: time saving

***Helpful: barrier: complicates task; driver/enabler: simplifies task

### Other

In 82% (162/198) of respondents, “recommendation by colleagues” was the deciding factor on which apps to install and use (supplementary tables 8). Additional features including perceived medical app accuracy and safety (supplementary table 9), app design experience (supplementary table 10), most useful medical app features (supplementary table 11), and themes for future app development (supplementary table 12) are listed in the supplementary tables.

## Discussion

This survey investigated mHealth use from a physician perspective in paediatric emergency care. The use of apps by individual physicians on their personal mobile devices in the absence of institutional devices was widespread. Nearly half of respondents reported using a formulary app daily, whereas instant messaging use was use by a third. The use of mobile devices and apps as part of patient management was generally well perceived by physicians.

### General considerations

The survey provided data from a large sample across a range of Emergency Department types, regions and subgroups of physicians involved in paediatric emergency care and the resuscitation of children. Respondents were nearly equally split between consultants and trainees. Whilst high use of mobile device technology as part of patient care has previously been reported from doctors in training [6, 28, 35], this study provides a more accurate overall reflection of reported use.

The provision of institutional mobile devices was low, forcing staff to default to personal devices, independent of whether they agree to this or not. Respondents showed a clear preference for institutional mobile devices for patient identifiable data such as smartphone photography, similar to existing literature [28, 36, 37]. Personal mobile devices were preferred for accessing medical apps (e.g. reference, formulary apps) for functions that did not require patient identifiable information [37].

Moving forward mobile device use policy and guidance needs to reflect this reality of widespread personal mobile device use in the clinical environment that has previously been reported [28, 37–39], in the absence of institutional devices. Personal mobile devices may actually be a low-cost solution to improve and revolutionise healthcare [37].

Respondents reported variability in provisioned Wi-Fi, with lack of Wi-Fi reported as a barrier to app use. Infrastructure provision such as free Wi-Fi and apps are key to support this advance in technology in healthcare [9, 10, 39–42].

There has been a lack of patient involvement in the development of digital strategies. In 2014 the iDoc project which provided junior doctors with medical apps on their mobile devices explored potential perceptions of apps. They proposed that app use at the bedside might be perceived as “using social media and playing games” or that patients “can see it negatively at the bedside” [28, 43, 44]. Physicians in our survey reported mHealth in general as an acceptable development, as previously reported [21]. In the experience and opinion of respondents to our survey, patients may perceive this use as acceptable as well. This opinion appears to be strengthened by taking the step of explaining mobile device use to patients. Similarly respondents reported consenting patients to smartphone photography and taking written consent for patient identifiable areas such as the face, reflecting guidance from the British Association of Dermatologists [45]. Our results support this pragmatic inclusive approach as part of good digital practice

### Medical apps

Half of our respondents used formulary apps, and one-third used medical calculator or clinical score apps daily. The single leading app was the *BNF/BNFc* formulary app; this app has consistently been chosen as the most popular and widely used medical app in the UK. Reported reasons include ease of use, ready access, access to standardised national guidance, and frequent updates [43, 46]. Others may wish to develop their own version of a free national formulary apps based on the UK experience. This approach may also serve as a blueprint when developing future medical apps.

The two leading resuscitation apps were *PaediatricEmergencies* [11] and *NeoMate* [47]. Developers have shown the superiority of apps in paediatric prescribing and burns management compared to paper-based resources [3, 5, 12].

Regulators classify apps as medical devices if they calculate medicine doses, diagnose disease, or provide a risk score [48, 49]. Clinicians currently using apps that perform these functions without regulatory approval need to be aware of the patient safety implications [48–51]. Regulatory approval can reassure both clinicians and patients, yet only two apps (*Mersey Burns* and *NeoMate*) reported in this survey are licensed [5]. In the UK potential issues can be reported to the regulator via the yellow card scheme (https://yellowcard.mhra.gov.uk/).

### Communication

Instant messaging use in healthcare is widespread in the UK and Ireland [38]. Convenience and ease of use in the absence of alternatives were the main reported driver. Respondents defaulted to consumer instant messaging apps despite being aware of security concerns [24, 38, 41, 42], as has been reported from other European countries [18–21, 52]. The NHS instant messaging guidance is based on the European GDPR Regulation [14–16] which recommends the use GDPR compliant secure messaging apps with AES 256 encryption. If these are not available, consumer instant messaging platforms can be used [34, 39].

The National Cyber Security Centre guidance recommends not allowing anyone else to use mobile device used for patient management, setting the device to lock out when not being used and requiring a passcode immediately, disabling message notifications on the device’s lock-screen, and enabling remote-wipe in case loss or theft [39, 45], e.g. by registering the device with the NHS BYOD scheme [42]. Other countries have developed similar approaches [24–26].

Some hospitals have banned instant messaging apps wholesale because of security and data governance concerns [38]. This is unlikely to be a long-term solution, and is difficult to enforce given advances in technology, absence of alternatives, and human behaviour [38]. However since the release of this survey, the National Trauma Network in the UK has approved *WhatsApp* as an official communications app in line with NHS Digital guidance [42, 53]. In this setting the use of *WhatsApp* revolves around team communication and team management in major incidents, rather than transmitting confidential patient information.

The breach of data protection policies when using messaging apps was raised as the leading data governance concern in our survey. This mainly concerned the sending and sharing of patient details and patient identifiable information e.g. images of ECGs or fractures and the subsequent storage of these on both the sender’s and the receiver’s device. This risk is increased if patient images are held on a personal device, even if anonymised, with an incumbent responsibility to delete any clinical images in an appropriate timeframe [38].

Respondents were especially concerned with data governance once send to other mobile devices. The presence of historic images with patient details on colleagues’ personal mobile devices was reported. One way to address this is the use of specialist secure communications app where images are not stored on the device, but on a secure server, such as *Siilo*, *MDSAS*, *Hospify*, *Forward* and *MedxNote*, used in the NHS [54, 55].

**Fig. 1:**
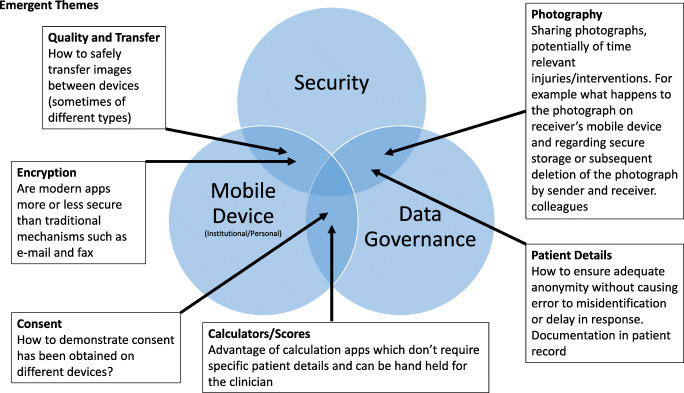
Emergent themes: Security, data governance and device type

In summary, staff have identified advantages in using mHealth. The use of personal devices is widespread. Concerns have been raised regarding data governance, especially when using instant messaging apps and smartphone photography, which can summarised in three key themes: security (data and device), data governance and device (personal versus institutional mobile device) (Fig. 1). Currently staff have found their own solutions to this issue, i.e. shared decision making with patients when using mobile device technology as part of consultation. Despite the existence of software solution to address these concerns such as secure messaging apps [54, 55] and registering the personal device with the NHS BYOD scheme [42], these are not widely adopted. Guidance needs to address this, so that staff can take advantage of the benefits of mHealth when using personals mobile device at work.

## Limitations

The survey was distributed through different channels including personal contact, colleges and societies, and social media; it is therefore impossible to calculate a response rate. Responses may have been influenced by self-selection of the respondents, the availability and support for certain devices and apps. The survey provided data across a range of emergency department types providing paediatric emergency care in the UK and Ireland and from a wide geographic spread across the UK and Ireland. This reduces the risk of bias, with results more likely to reflect a true snapshot of current practice.

## Conclusion

App use on the personal mobile devices, in the absence of access to institutional devices, was widespread, especially the use of a national formulary app. Instant messaging and smartphone photography were less common. Staff have adopted a pragmatic inclusive approach to data governance. Guidance needs to keep pace with this rapidly evolving technology. A strategic decision has to be made to either provide staff with institutional devices or use software solutions to address data governance concerns when using the personal device.

## Supplementary Information

ESM 1 (DOCX 47 kb)

ESM 2 (DOCX 61 kb)
