# Supplementary material for: A survey of mHealth use from a physician perspective in paediatric emergency care in the UK and Ireland
Source: Eur J Pediatr. 2021 Mar 25;180(8):2409–18. doi: 10.1007/s00431-021-04023-0 (PMC8285308; doi:10.1007/s00431-021-04023-0)
Supplement: Supplementary file 1 — (DOCX 47 kb) [file 431_2021_4023_MOESM1_ESM.docx]

# Appendix: Survey Questionnaire

1. Do you resuscitate children?
   1. Yes
   2. No
2. Are you a medical doctor
   1. Yes, I am a medical doctor
   2. No, other health care professional
3. Do you work in the UK or Ireland?
   1. Yes
   2. No
4. Could you select your region from below
   1. England
   2. Northern Ireland
   3. Scotland
   4. Wales
   5. Republic of Ireland
5. What is your gender
   1. Female
   2. Male
   3. Other
6. How old are you
   1. Number (free textbox)
7. Do you work at a PERUKI site
   1. Yes, I work at a PERUKI site
   2. No, I do not work at a PERUKI site
8. What is your role?
   1. Consultant (or equivalent on specialist register)
   2. General Practitioner (or equivalent on specialist register)
   3. Trainee grade doctor (senior house officer, specialist registrar or equivalent)
9. What is your specialty?
   1. Select specialty from list
10. Does your institution provide free Wi-Fi or internet access to use Apps at work (both on the institutional and your personal mobile devices)?
    1. Yes
    2. No
    3. Other (free textbox)
11. Are you provided with an INSTITUTIONAL device to run Medical Apps?
    1. Yes
    2. No
12. Are you aware of your institution's mobile device policy?
    1. Yes
    2. No
13. In relation to the following types of Apps and mobile device functions, please indicate how often you use them to help you with your clinical and educational activities: Select all relevant
    1. medication formulary or drug reference
    2. clinical score systems or medical calculator
    3. disease diagnosis or management
    4. procedure documentation
    5. CPD (Continuing Professional Development) or eportfolio
    6. education (revision & learning)
    7. calendar, rota
    8. password storage (login details)
    9. email access (work email)
    10. staying in touch with colleagues
    11. web access
14. Do you use Medical Apps on your PERSONAL mobile device?
    1. Yes
    2. No
15. Which type of mobile device do you use?
    1. Blackberry
    2. iPhone/iPad
    3. Android Phone/Tablet
16. How many Medical Apps do you have on your PERSONAL mobile device (smartphone) per device?
    1. 0
    2. 1-5
    3. 6-10
    4. more than 10
17. Which Medical Apps do you have on your own PERSONAL device (e.g. electronic formulary, drug dose calculators, clinical guidelines, etc)?
    1. textbox
18. Are you aware of any patients that has suffered harm, due to the use of medical apps as part of their care (e.g. wrong drug dose, etc.)?
    1. Yes
    2. No
19. How do you perceive the use of Mobile Devices and Medical Apps during consultation as a medical professional?
    1. Rude
    2. Unprofessional
    3. shows lack of knowledge
    4. Acceptable
    5. Professional
    6. Other
20. How do think patients and relatives perceive the use of Mobile Devices and Medical Apps during consultation?
    1. Rude
    2. Unprofessional
    3. shows lack of knowledge
    4. Acceptable
    5. Professional
    6. Other
21. Have you had any comments by patients or their relatives regarding Medical Apps use in the Clinical Environment?
    1. Textbox
22. Do you have any concerns regarding patient confidentiality when using Medical Apps?
    1. No
    2. May be
    3. Yes
23. Could you comment on any specific patient confidentiality concerns when using Medical Apps.
    1. textbox
24. Which mobile device (personal or institutional) do you prefer to use?
    1. Referring a patient
    2. Medical Apps
    3. Taking photograph of Burn, Rash, Laceration, etc.
25. How often do you use the following mobile device functions when seeking advice regarding patient management?
    1. Text (SMS) messaging
    2. Smartphone Photography
    3. Instant Messaging
    4. Secure Specialist Medical Messaging App
26. What is your reason for using smartphone photography and messaging Apps to communicate with colleagues?
    1. I only use them on the INSTITUTIONAL Device
    2. No alternative provided by my institution (e.g. NHS)
    3. Convenient & ease of use
27. When using smartphone photography and messaging Apps, do you anonymise patient details?
    1. Sent information without any patient identifier
    2. Sent, but omit key details (e.g. provide only initials or bed space)
    3. It's end to end encrypted so I don't
28. Have you encountered any barriers regarding the use of Medical Apps in the Clinical Environment? Select all relevant
    1. Colleagues
    2. Institution
    3. Patients
    4. Technical Issues
    5. Internet or Wi-Fi Connection to run Medical App
    6. Price (not free to download)
    7. Takes to much time to use Medical Apps
    8. To complicated to use Medical Apps
    9. Other (please specify)
29. What has enabled the use of Medical Apps in the Clinical Environment in your setting? Select all relevant
    1. Colleagues
    2. Institution
    3. Patients
    4. Technical Issues
    5. Internet or Wi-Fi Connection to run Medical App
    6. Price (free to download)
    7. Medical Apps speed up processes e.g. checking drug doses, etc
    8. Medical Apps simplify processes e.g. prescribing infusions, etc
    9. Other (please specify)
30. How do you know that a Medical App is accurate (e.g. gives the right drug dose for weight and age in a child)? Select all relevant
    1. I assume the Medical App is accurate as is downloaded from the android/apple/etc store
    2. I assume the Medical App is accurate if it is accredited by a trusted body (eg Royal College)
    3. I know the developer
    4. I have checked and tested the App (eg tested the drug dose calculator)
    5. I do not know, but use them anyway
    6. Recommended by someone I trust
    7. unable to comment
31. How do you know that a Medical App is safe (does not contain any bugs or viruses)? Select all relevant
    1. The App is safe as I downloaded it from the android/apple/etc store
    2. The App is safe as it is accredited by a trusted body (eg Royal College, etc)
    3. I know the developer
    4. I have checked and tested the App
    5. I do not know, but use them anyway
    6. Recommended by someone I trust
    7. unable to comment
32. How do you select a Medical App for installation on your device? Select all relevant
    1. Trial and error
    2. Recommendation by colleagues
    3. App accredited by institution (eg Royal College, NHS institution or other government institution)
    4. Recommendation from scientific journal, eg EMJ
33. Have you written or designed an App?
    1. I have written a number of Apps
    2. I have written one App
    3. I have attempted to write one App
    4. I have no idea how this work
34. What features do you find most useful in a Medical App?
    1. textbox
35. What type of Medical Apps would you like to see in the future in the Emergency Department?
    1. textbox
