# Supplementary material for: A survey of mHealth use from a physician perspective in paediatric emergency care in the UK and Ireland
Source: Eur J Pediatr. 2021 Mar 25;180(8):2409–18. doi: 10.1007/s00431-021-04023-0 (PMC8285308; doi:10.1007/s00431-021-04023-0)
Supplement: Supplementary file 2 — (DOCX 61 kb) [file 431_2021_4023_MOESM2_ESM.docx]

# Appendix: Supplementary tables

1 Supplementary table: Respondents place of work

| Place of Work | Type of Hospital | n=198 | % |
| --- | --- | --- | --- |
| - England |  | 129 | 65% |
| Alder Hey Children's Hospital | University | 10 | 5% |
| Barts & The London | University | 2 | 1% |
| Birmingham Children's Hospital | University | 2 | 1% |
| Bristol Royal Hospital for Children | University | 5 | 3% |
| Chelsea & Westminster Hospital | University | 1 | 1% |
| Derriford hospital | University | 0 | 0% |
| Eveline Children’s Hospital | University | 0 | 0% |
| Frenchay Hospital | University | 0 | 0% |
| Hull Royal Infirmary | University | 5 | 3% |
| Ipswich Hospital | DGH | 2 | 1% |
| King's College Hospital | University | 1 | 1% |
| Leeds General Infirmary | University | 3 | 2% |
| Leicester Royal Infirmary | University | 6 | 3% |
| Lewisham Hospital | University/DGH | 3 | 2% |
| North Manchester General Hospital | University | 0 | 0% |
| Nottingham Children's Hospital | University | 4 | 2% |
| Ormskirk Hospital | DGH | 1 | 1% |
| Queen Alexandra Hospital | University | 1 | 1% |
| Queen Elizabeth Hospital, Kings Lynn | DGH | 1 | 1% |
| Royal Alexandra Children's Hospital | University | 4 | 2% |
| Royal Derby Hospital | University | 6 | 3% |
| Royal Devon and Exeter Hospital | University | 0 | 0% |
| Royal Free Hospital | University | 0 | 0% |
| Royal Manchester Children's Hospital | University | 1 | 1% |
| Royal United Hospital | DGH | 1 | 1% |
| Salford Royal Hospital | University | 0 | 0% |
| Sheffield Children's Hospital | University | 1 | 1% |
| St George's Hospital | University | 2 | 1% |
| St Mary's Hospital | University | 2 | 1% |
| Sunderland Royal Hospital | University | 7 | 4% |
| University Hospital Southampton | University | 7 | 4% |
| Watford General Hospital | DGH | 4 | 2% |
| Not recorded |  | 47 | 24% |
| - Northern Ireland |  | 27 | 14% |
| Royal Belfast Hospital for Sick Children | University | 18 | 9% |
| Not recorded |  | 9 | 5% |
| - Scotland |  | 19 | 10% |
| Aberdeen Royal Infirmary | University | 3 | 2% |
| University Crosshouse Hospital | University/DGH | 0 | 0% |
| Forth Valley Royal Hospital | DGH | 6 | 3% |
| Royal Hospital for Sick Children - Edinburgh | University | 6 | 3% |
| Royal Hospital for Sick Children - Glasgow | University | 1 | 1% |
| Not recorded |  | 3 | 2% |
| - Wales |  | 13 | 7% |
| Children's Hospital for Wales | University | 10 | 5% |
| Morriston Hospital | University/DGH | 1 | 1% |
| Not recorded |  | 2 | 1% |
| - Republic of Ireland |  | 10 | 5% |
| Cork University Hospital | University | 5 | 3% |
| Our Lady's Children's Hospital | University | 1 | 1% |
| Tallaght Children's Hospital | University | 1 | 1% |
| Not recorded |  | 3 | 2% |
| Site response rate based on physicians who recorded site they worked at: 82%, (physicians from 37 out of a total of 45 PERUKI site responded to the survey), response per site median 2, mean 3 (where PERUKI site was recorded). | | | |

2 Supplementary table: Speciality of respondents

| Specialty of respondents | n=197 | % |
| --- | --- | --- |
| Paediatrics  Paediatrics & Paediatric Emergency Medicine (subspecialty training: 20% (40/197) | 123 | 62% |
| Emergency Medicine  (Emergency Medicine & Paediatric Emergency Medicine: 11% (22/197) | 57 | 29% |
| Intensive Care & Anaesthetics | 10 | 5% |
| Surgical and other specialties | 9 | 5% |

3 Supplementary table: Patients comments on app use during consultations

| Theme | Responses (n=138) |
| --- | --- |
| No comments | 89 had had not comments by patients regarding medical app use  28 thought the reason why they never had any comments was, that they always explain app use to patients prior to use |
| Complains regarding medical app use | 7 reported that patients complained regarding medical app use. |
| Compliments regarding medical app use | 7 stated that they had compliments from patients when using medical apps |
| Do not use medical apps in front of patients | 5 reported that they do not use medical apps in front of patients |
| Mixed reviews | 2 reported mixed reviews |

4 Supplementary table: Patient confidentiality concerns

| Theme | Response, n=66 |
| --- | --- |
| Data security | 14 respondents had data security concerns ranging from the hacking, to security of sending and receiving device, to encryption despite one respondent highlighting that instant messaging encryption standards are higher than more traditional methods of communication. |
| Colleagues | 13 respondents had concerns regarding colleagues use of smartphone photography and instant messaging apps, especially with regards to colleagues not anonymising patient details, sending information to a wrong recipient and images being shared with unauthorised personal. |
| Data Governance | 13 respondents had data governance concerns ranging from the use of none NHS approved apps, patient details being sent on a non-NHS network, to colleagues having historic images on their mobile devices. |
| Apps | 11 responded had specific app concerns ranging from use of MDSAS app, institutional messaging apps, to the use whatsapp for team communication only, and discouraging whatsapp use to communicate patient identifiable information, no concerns for medical calculator apps that do not use patient identifiable information. |
| Do not use | 12 respondents stated that they do not use apps or only apps that do not require patient identifiable information or not allowed to use smartphone photography |
| Smartphone photography | 8 had concerns regarding smartphone photography, especially with regards to images of children. One respondent highlighted that photographs can be time and date stamped. |
| Anonymisation | 7 respondents had concerns regarding messages where not anonymised and patient identifiable information was freely shared. |
| Guidance | 4 respondents requested expert guidance for photography, documentation of instant messaging conversations, including guidance from the regulatory body. |
| Consent | 3 respondents had concerns regarding the appropriate consent being taken. |
| No concerns | 3 respondents had no concerns |

4 Supplementary table: Frequency of messaging app and smartphone photography use when seeking patient management advice

|  | Total use (n=198, %) | Frequency | | |
| --- | --- | --- | --- | --- |
|  |  | Once  (n=198, %) | Monthly  (n=198, %) | Weekly  (n=198, %) |
| Instant Messaging (e.g. What’s App, etc) | 76, 38%, | 11, 6% | 25, 13% | 40, 20% |
| Secure specialist Messaging App (e.g. Siilo, etc) | 12, 6% | 3, 2% | 2, 1% | 7, 4% |
| Text (SMS) messaging | 56, 29% | 9, 5% | 18, 9% | 29, 15% |
| Smartphone Photography | 57, 29% | 19, 10% | 26, 13% | 12, 6% |

###

5 Supplementary table: Use of anonymisations when communicating patient identifiable information of respondents using messaging apps or smartphone photography

|  | Total: Anonymise patient identifiable information  (n=198, %) | Level of anonymisation | | Do not anonymise, as it is end-to-end encrypted  (n=198, %) |
| --- | --- | --- | --- | --- |
|  |  | Anonymise: Sent information without any patient identifier  (n=198, %) | Anonymise: Sent, but omit key details (e.g. provide only initials or bed space)  (n=198, %) |  |
| Messaging apps | 57, 29% | 35, 18% | 22, 11% | 8, 4% |
| Smartphone  photography | 57, 29% | 50, 25% | 7, 3% | 6, 3%, |

6 Supplementary table: Barriers and enablers of app use

| Barrier, n=26 | Enabler, n=11 |
| --- | --- |
| - Poor phone and Wi-Fi reception, default to personal 4G - Use in front of patients - Mobile device issues e.g. small screen size, computer illiterate - No barriers - App issues, e.g. login - Not required - Hand hygiene - Governance (lack of support) - Cost | - Pharmacist recommendation (BNF/BNFc app) - Cognitive off-loading - Education - Designed by consultants in department & encouraging use - Part of standard care (mobile device use for taking observations) |

7 Supplementary table: Method employed to select app to download

| Selection Method | n=198, % |
| --- | --- |
| Trial and error | 60, 30% |
| Recommendation by colleagues | 162, 82% |
| App accredited by institution (eg Royal College, etc.) | 124, 63% |
| Recommendation from scientific journal | 76, 38% |

8 Supplementary table: Accuracy (right drug dose) and safety (no software viruses or bugs) of app

|  | Accuracy  n=198, % | Safety  n=198, % |
| --- | --- | --- |
| I assume the Medical App is accurate as is downloaded from the android/apple/etc store | 32, 16% | 59, 30% |
| I assume the Medical App is accurate if it is accredited by a trusted body (e.g. Royal College) | 113, 57% | 61, 31% |
| I know the developer | 28, 14% | 17, 9%, |
| I have checked and tested the App (e.g. tested the drug dose calculator) | 78, 39% | 29, 15% |
| I do not know, but use them anyway | 6, 3% | 53, 27% |
| Recommended by someone I trust | 40, 20%, | 30, 15% |
| Unable to comment | 25, 13%, | 35, 18% |

10 Supplementary table: App design experience

|  | n=198, % |
| --- | --- |
| I have written a number of Apps | 1, 1% |
| I have written one App | 12, 6% |
| I have attempted to write one | 18, 9% |
| I have no idea how this works | 160, 81%, |

12 Supplementary table: Most useful feature of medical app

|  | Theme | n=198 |
| --- | --- | --- |
|  |  | |
| App Design | Clear user interface | 26 |
|  | Ease of use | 25 |
|  | Speed | 13 |
|  | Offline | 6 |
|  | Search function | 5 |
|  | Single purpose | 1 |
|  | Large text and buttons | 1 |
|  |  | |
| App Topic | Reference/aid memoire | 12 |
|  | Calculator | 8 |
|  | How to make up infusions –  calculation needs to be shown to allow checking | 5 |
|  | Relevant to clinical practice | 6 |
|  | Formulary | 3 |
|  |  | |
| Other App Features | Regularly updated | 8 |
|  | Endorsed | 3 |
|  | Reasonable price (free) | 2 |
|  | Point of care | 1 |
|  | Work on different platforms | 1 |
|  | Added Reality in SIM | 1 |

12 Supplementary table: Future app development

| Theme | N=198 | Topic |
| --- | --- | --- |
| AI (Artificial Intelligence) Decision Tools | 2 |  |
| Communication | 9 | - Bleep filtering - Referral |
| CPD (continuous professional Development) | 4 | - Procedure Log |
| Distraction App | 1 |  |
| ED Tracking | 1 |  |
| EMR (Electronic Medical Record) | 8 | - Dictation - Patient held EMR - Patient held ECG/Medical Images |
| EWS (Early Warning Score) | 1 |  |
| Formulary | 15 | - Calculator (e.g. DKA) - Infusion - Microbial Guidance |
| Guidelines/Reference | 46 | - ALSG Accreditation - Best Practice - BP (Blood Pressure) Centiles - Burns - Dermatology - Differential Diagnosis - ECG - Fracture - Local Guidance - Minor Injuries - Minor Injuries - Paediatric - Paediatric - Medical - Paediatric - Radiology - Procedures - Rare Condition Management - Safe Discharge Checklist - Trauma – Head Injuries - Trauma Paediatric - Toxicology |
| Patient Advice Leaflets | 4 |  |
| Research | 1 |  |
| Resuscitation | 17 | - APLS - Calculator - Critical Care - Retrieval - Timing Prompts |
| Self-Harm Questionnaire (Teenager) | 1 |  |
| Simulation | 2 |  |
| Task Prioritisation | 1 |  |
| Triage - Trauma | 1 |  |
| Unknown/None | 2 |  |
